# Transcriptomic survey reveals multiple adaptation mechanisms in response to nitrogen deprivation in marine *Porphyridium cruentum*

**DOI:** 10.1371/journal.pone.0259833

**Published:** 2021-11-18

**Authors:** Li Wei, Wuxin You, Zhengru Xu, Wenfei Zhang

**Affiliations:** 1 Ministry of Education Key Laboratory for Ecology of Tropical Islands, Key Laboratory of Tropical Animal and Plant Ecology of Hainan Province, College of Life Sciences, Hainan Normal University, Haikou, China; 2 Department of Plant Biochemistry, Ruhr University Bochum, Bochum, Germany; 3 College of Foreign Language, Hainan Normal University, Haikou, China; Bharathidasan University, INDIA

## Abstract

Single-cell red microalga *Porphyridium cruentum* is potentially considered to be the bioresource for biofuel and pharmaceutical production. Nitrogen is a kind of nutrient component for photosynthetic *P*. *cruentum*. Meanwhile, nitrogen stress could induce to accumulate some substances such as lipid and phycoerythrin and affect its growth and physiology. However, how marine microalga *Porphyridium cruentum* respond and adapt to nitrogen starvation remains elusive. Here, acclimation of the metabolic reprogramming to changes in the nutrient environment was studied by high-throughput mRNA sequencing in the unicellular red alga *P*. *cruentum*. Firstly, to reveal transcriptional regulation, de novo transcriptome was assembled and 8,244 unigenes were annotated based on different database. Secondly, under nitrogen deprivation, 2100 unigenes displayed differential expression (1134 upregulation and 966 downregulation, respectively) and some pathways including carbon/nitrogen metabolism, photosynthesis, and lipid metabolism would be reprogrammed in *P*. *cruentum*. The result demonstrated that nitrate assimilation (with related unigenes of 8–493 fold upregulation) would be strengthen and photosynthesis (with related unigenes of 6–35 fold downregulation) be impaired under nitrogen deprivation. Importantly, compared to other green algae, red microalga *P*. *cruentum* presented a different expression pattern of lipid metabolism in response to nitrogen stress. These observations will also provide novel insight for understanding adaption mechanisms and potential targets for metabolic engineering and synthetic biology in *P*. *cruentum*.

## Introduction

Nitrogen is one of all essential nutrient components for photosynthetic organisms [1]. In various ecosystems, including terrestrial and aquaculture environments, nitrogen is one of the most important nutrients and affects the growth of green plants, algae and cyanobacteria [2]. It is reported that nitrogen deficiency of plant induces changes in many physiological processes including weakened photosynthesis, altered carbon partition, changed chlorophyll and carotenoid biosynthesis, and reduced growth etc. In a majority of microalgae, lipid and polysaccharide productions as a carbon storage are often increased under nitrogen stress [3]. For instance, both triacylglycerol (TAG) and starch accumulate were influenced by nitrogen availability in the green microalga *Chlamydomonas reinhardtii* [4], *Chlorella sorokiniana* [5], diatom *Phaeodactylum tricornutum* [6] and red alga *Cyanidioschyzon merolae* [7]. In some oleaginous microalgae such as *Nannochloropsis* [8] and *Neochloris oleoabundans* [9], TAG production is largely enhanced under nitrogen starvation. Thus, under the lack of nitrogen, photosynthetic organisms would have to reprogram their biosynthetic, metabolic and/or transcriptional events to adapt to new environment.

*Porphyridium* spp. as a group of the unicellular red algae belongs to the *Plantae*, the *Biliphyta*, the *Rhodophyta*, the *Proteorhodophytina*, the *Porphyridiophyceae*, the *Porphyridiales*, the *Porphyridiaceae* [10, 11]. *Porphyridium* spp. can naturally accumulate many high value products with bioactive substances, including phycoerythrin (PE) [10] and extracellular polysaccharides [12], which can be used in the fields such as medicine, nutrition and food [11]. Additionally, the red microalgae *Porphyridium* spp. is considered a potential source for these three long-chain polyunsaturated fatty acids (LC-PUFAs), due to its high biomass production [13] and abundant EPA (eicosopentaenoic), ARA (Arachidonic acid), and LA (linoleic acid) contents [13]. Although *Porphyridium* is a valuable alga resource for industrial application, majority of studies to date have focused on species culturing optimization [10], ecological function [14], pharmaceutical and food processing (Bioactive substances of *Porphyridium* and their applications), phylogenetic evolution [15] and substance (PE or EPA) extraction treatment. Notably, researchers have found that the contents of LC-PUFA and PE in *Porphyridium* are highly dependent on environmental conditions such as nitrogen stress [16]. In *Porphyridium purpureum*, under nitrogen limitation condition, their growth, photosynthesis, and chlorophyll content (40% decrease) were significantly reduced and respiration was increased [17]. In *Porphyridium cruentum*, the photochemical efficiency and the activity of photosystem II (PSII) reaction centers showed a significantly decreased trend (15%), which implied PSII would occur photoinhibition under nitrogen starvation [17]. Moreover, compared to green and yellow-green algae, red algae might employ different metabolic adaption to cope with nitrogen starvation [16, 18–20]. Thus, many studies demonstrated that photosynthesis, lipid accumulation, and fatty acid biosynthesis would be influenced by nitrogen starvation in *P*. *cruentum*. However, the knowledge of the molecular mechanism for stress adaptation remains limited under nitrogen deficiency. Hence, it is necessary to perform some molecular insight studies in *P*. *cruentum*.

In this study, we aimed to interrogate transcriptomic change of *P*. *cruentum* after the shift from nitrogen repletion to nitrogen depletion by high quality mRNA-Seq data using Illumina NextSeq6000 technology. Firstly, *de novo* transcriptome was assembled and the trimmed reads were processed to yield unigenes. Annotated unigenes were analyzed by GO terms and KEGG pathways. The result showed that 8,244 unigenes were firstly annotated in *P*. *cruentum*. These investigations provided a global insight of the cells at the RNA level. Next, we tried to interrogate globe response after *P*. *cruentum* cells exposed to nitrogen depletion environment. By the analysis of differential gene expression, 1134 and 966 unigenes were up- and down- regulated, respectively. For predicting the potential mechanisms of differential expression, enriched GO terms and KEGG pathways were elucidated. Some pathways including carbon/nitrogen metabolism, photosynthesis, and lipid metabolism would be reprogrammed in *P*. *cruentum*. The result demonstrated that nitrate assimilation was enhanced and photosynthesis was impaired. Compared to other green algae, red microalga *P*. *cruentum* presented a different expression pattern of lipid metabolism in response to nitrogen stress. This is not only the first report of transcriptome from *P*. *cruentum*, but also the first understanding of adaptation mechanisms after nitrogen deprivation in the single-cell red microalgae. Current study would provide a novel insight into underlying adaption mechanisms in response to nitrogen deprivation and open new avenue for the future synthetic biology and applied studies in microalgae.

## Results and discussion

### Physiological changes in response to nitrogen deprivation

**Fig 1:**
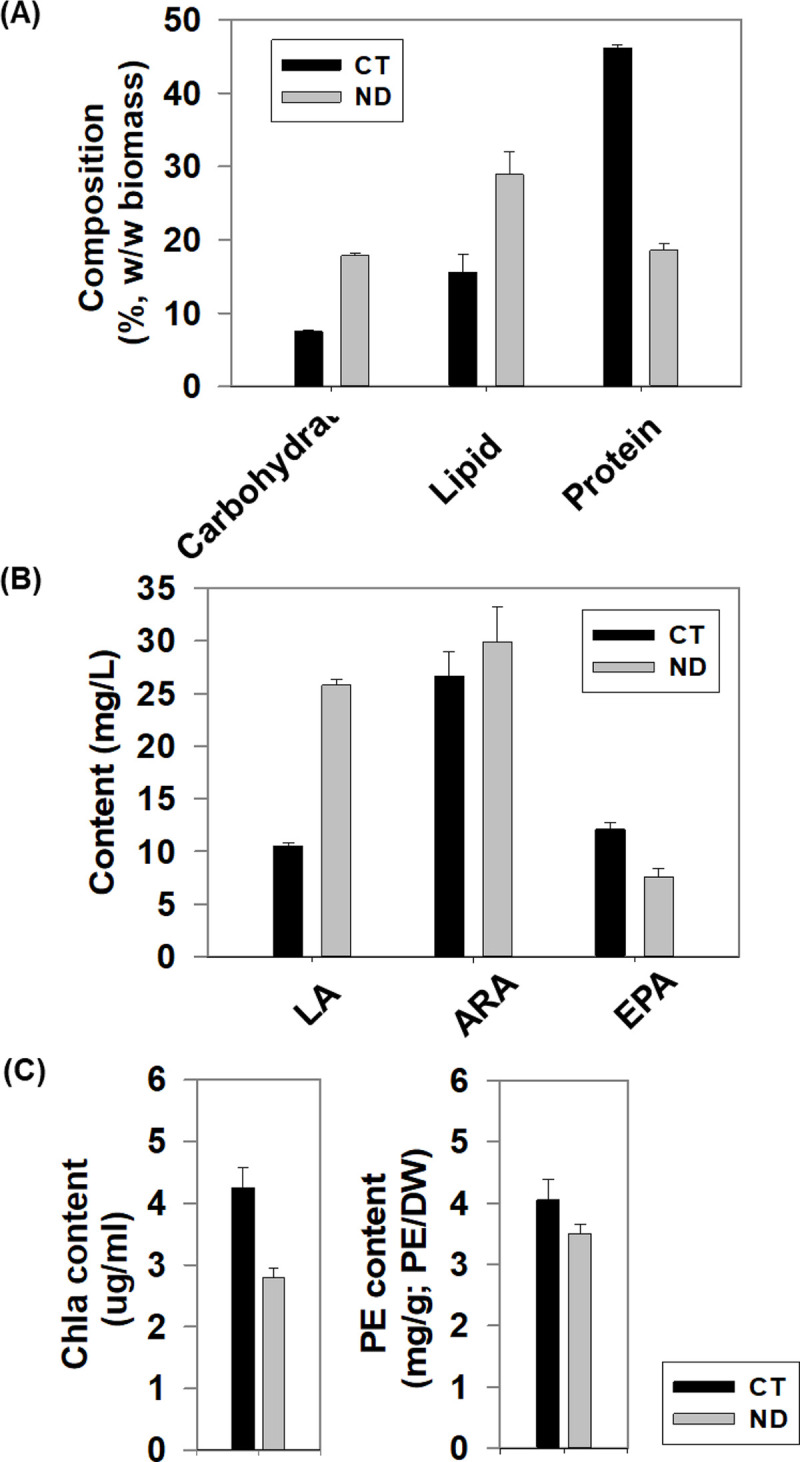
Physiological measurements of *P*. *cruentum* under CT and ND. (**A**) The of biochemical compositions of lipid, carbohydrate and protein at 72h under ND and under CT. (**B**) The contents of three key LC-PUFAs were detected. (**C**) The contents of *Chla* and PE were compared under CT and ND.

To identify the change of lipid, carbohydrate, protein and delineate their interactions, the physiological responses were tracked under two contrasting culture conditions for *P*. *cruentum* (strain GY-H56): nitrogen repletion (CT: control) and nitrogen deprivation (ND). On the third day under nitrogen deprivation, the contents of carbohydrate and lipid were increased 78% and 45% compared to nitrogen repletion, respectively (**Fig 1A**). However, the content of protein showed a 48% decrease. As for LC-PUFAs, we observed that LA and ARA were significantly increased under ND condition, while EPA was decreased (**Fig 1B**). Additionally, the contents of *Chla* and PE were also measured, the result showed that the content of *Chla* and PE were reduced under ND (**Fig 1C**).

#### *De novo* assembly of *P*. *cruentum* transcriptome data from Illumina sequencing

To reveal the adaptation of *P*. *cruentum* in response to nitrogen starvation, we cultivated red microalga *P*. *cruentum* cells under nitrogen repletion (as control; CT) and depletion (as treatment; ND), and then constructed four cDNA libraries (yielded from two biological replicates with high correlation (99%)). Using Illumina NextSeq6000 sequencing technology, each reaction can yield 2 × 150 bp independent reads from either end of a DNA fragment. In total, there were 66,239,986 byte raw reads (from CT and ND samples) obtained and the clean reads (65,385,802 bytes) accounted for more than 98% after the raw reads trimmed (**Table 1**). After stringent quality filtering, high-quality reads were exhibited 98.06%-98.53% Q20 bases (94.07%-95.40% Q30) and a GC value of 57.13% (**Table 1**). This result demonstrated that the data are high-quality. A total of 8,244 unigenes were successfully assembled using the Trinity software [21], with a mean length of 1,944 bp and an N50 of 3,072 bp. Among all the assembled unigenes, 1,876 of which (approximately 23%) were less than 500 bp, and 1,749 unigenes (22%) were longer than 3000 bp, whereas most of the unigenes (4,619) (65%) ranged from 500 to 3000 bp (**S1 Table and S1 Fig**). To further interrogate the quality of identified unigenes, *de novo* assembled *P*. *cruentum* trancriptome by trinity software were taken as a reference se. All the yielded clean reads were mapped to the assembled unigenes by RSEM software. The number of reads mapped to each unigene analysis uncovered that 3,958 unigenes (about 48%) and 1,814 unigenes (approximately 22%) consist of more than 100 and 1000 reads each, respectively, and only a few unigenes (3.3%) were originated from less than 10 reads. Correlation coefficients between our biological replicates (Kendall τ) were on average 0.99, and agreement between those replicates was good, making the repeatability suitable for further analysis (**S2 and S3 Figs**). Moreover, qPCR was performed to validate gene expression (**S4 Fig**). The *P*. *cruentum* transcriptome shotgun assembly has been deposited at NCBI/Bioproject under the number of PRJNA680008 and SRA number (SRR13167200, SRR16764428, SRR16764434, SRR16764430.

**Table 1:** Summary of transcriptome sequencing data of *P*. *cruentum* under control (CT) and nitrogen deprivation (ND).

Sample	Raw reads	Raw bases	Clean reads	Clean bases	Error rate (%)	Q20 (%)	Q30 (%)	GC content (%)
CT_1	14973130	2260942630	14744430	2214991444	0.0238	98.53	95.4	57.13
CT_2	15595830	2354970330	15423360	2315543781	0.025	98.06	94.07	57
ND_1	17647004	2664697604	17473994	2627674200	0.0241	98.42	95.09	56.84
ND_1	18024022	2721627322	17744018	2665207957	0.0241	98.41	95.08	56.87

Q20: The percentage of bases with a Phred value > 20. Q30: The percentage of bases with a Phred value > 30.

### Functional annotation and classification

**Fig 2:**
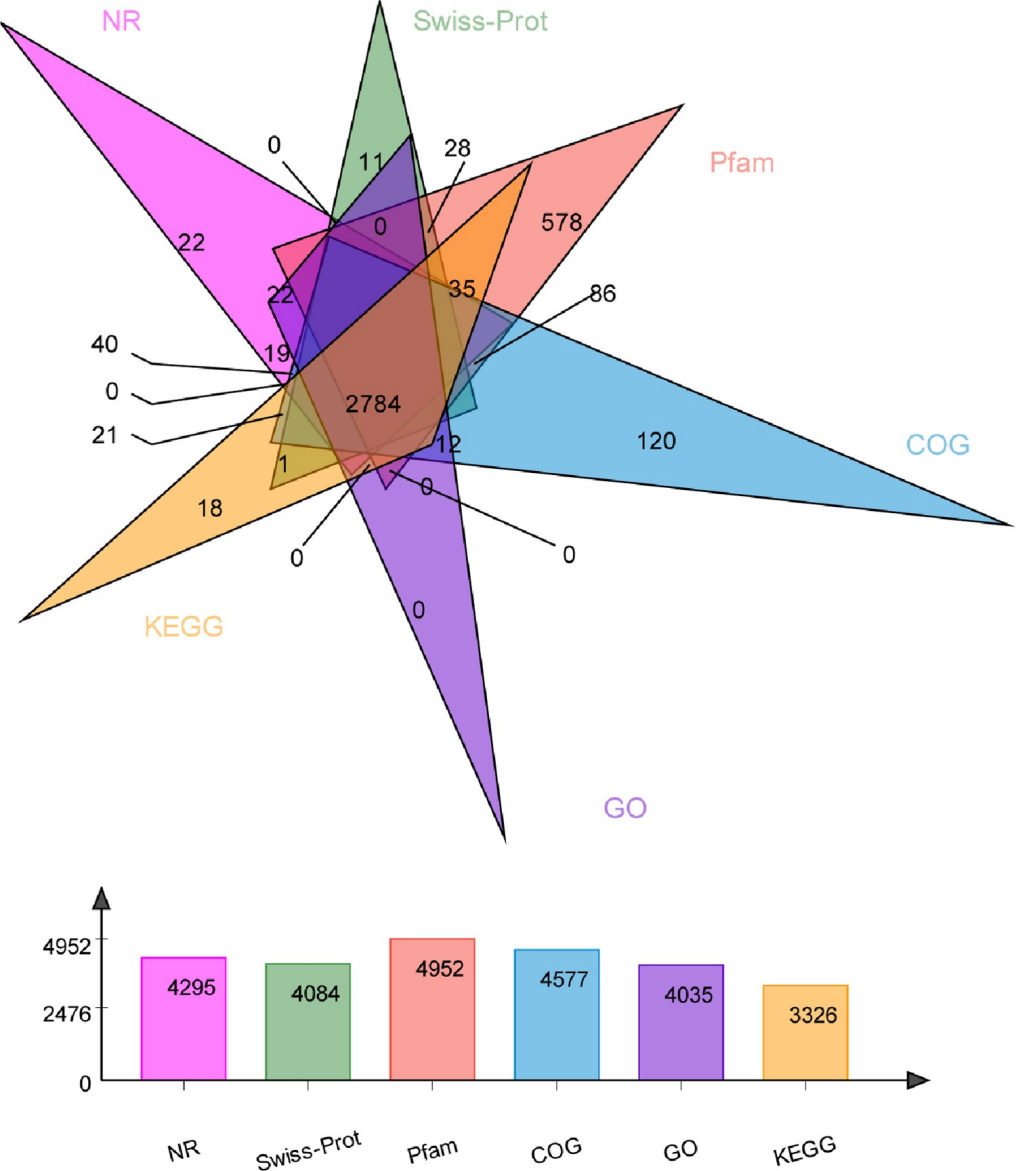
Venn diagram and number of annotated unigenes using different database. Below database was used to annotate unigenes. Nr: NCBI’s non-redundant database. GO: Gene Ontology. EC number and KO: The annotation results of KEGG (Kyoto Encyclopedia of Genes and Genomes) database. COG: Clusters of Orthologous Groups (COG) database. Swiss-Prot: A manually annotated and reviewed protein sequence database. Pfam: Protein family. Each figure represents the number of annotated unigenes in corresponding databases.

**Fig 3:**
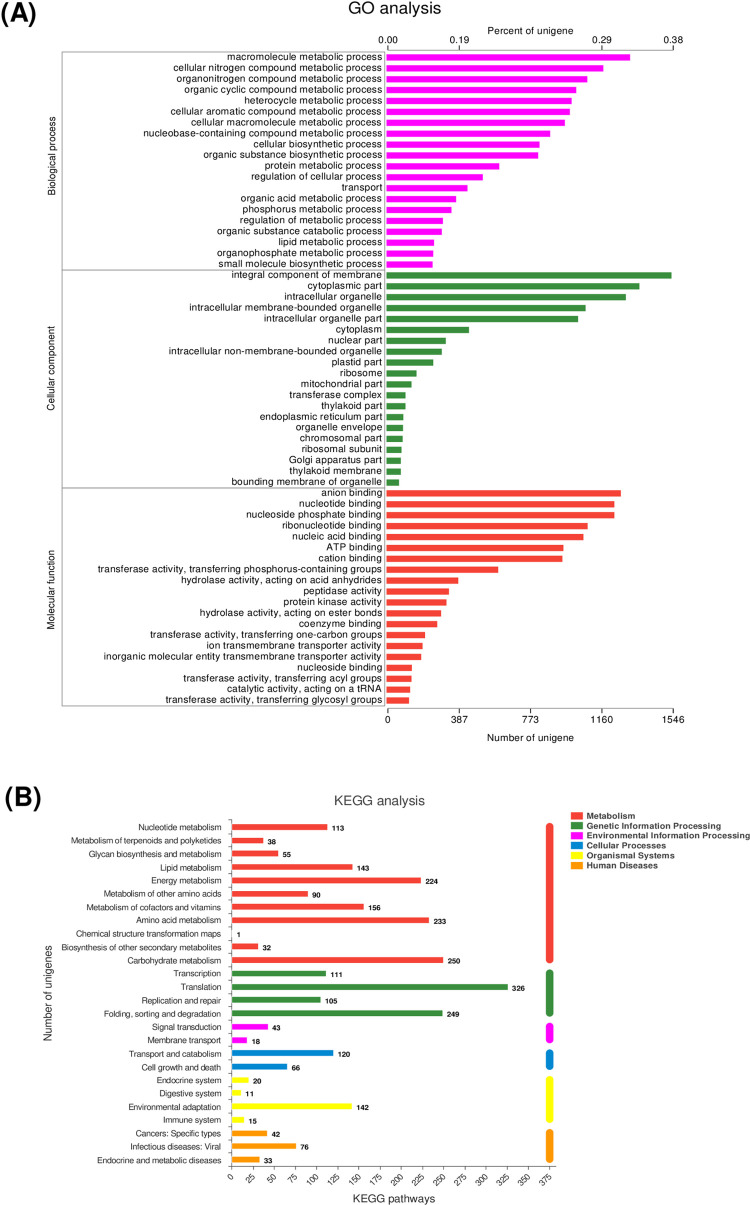
Annotation enrichments and classfications of assembled core unigenes in *P*. *cruentum*. (**A**) Histogram of assembled unigene classfications against Gene ontology (GO). The *P*. *cruentum* unigenes were systematically classified into three subcategories by GO analysis, including biological process (BP), cellular component (CC), and molecular function (MF). Each bar represents the number of unigenes under each subcategory. (**B**) Pathway enrichment analysis of DEGs by KEGG.

To achieve the primary genetic information of *P*. *cruentum*, by NCBI BLAST tools, all assembled *P*. *cruentum* unigenes were performed to blast against NR database, SwissProt, Pfam, GO, KEGG and COG databases for functional annotation (**Fig 2**). All of the unigenes were annotated to genes with known functions in the above-mentioned databases based on the similarity of their sequences. Among the 8,244 unigenes, the greatest number of matches were annotated in the Pfam databse (4980 unigenes, 60.41% of all unigenes), followed by Nr (4317 unigenes, 52.37% of all unigenes), Swiss-Prot (4105 unigenes, 49.79% of all unigenes) and GO (4056 unigenes, 49.2% of all unigenes) (**Table 2 and Fig 2**). In total, 8,244 unigenes displayed homologous matches in at least one database and 2,764 (33%) unigenes were annotated in all six databases (**Fig 3A**). Species distribution of the total homologous sequences was calculated with match (with a cut-off E-value of 1.0E-5) in NR database (**S4 Fig**).

**Table 2:** Overview of the mRNA sequencing and assembly in *P*. *cruentum*.

Type	Unigene	Transcript
Total number	8244	9298
Total base	16030881	18003464
Largest length (bp)	17349	17349
Smallest length (bp)	201	201
Average length (bp)	1944.55	1936.27
N50 length (bp)	3072	3068
E90N50 length (bp)	3450	3433
Mean mapped percent (%)	96.201	97.585
GC percent (%)	56.22	56.18
TransRate score	0.42819	0.43115
BUSCO score	11.7% (0.8%)	11.7% (0.8%)

As above-mentioned, a total of 4,056 (49%) unigenes were successfully annotated by GO. These annotated unigenes were classified into three ontologies, including BP (biological process), CC (cellular component) and MF (molecular function). The distribution of unigenes was displayed in **Fig 3A**, these 4,056 unique sequences generated three terms, including 44% CC terms, 38% ME terms and 16% BP terms. In depth analysis into CC terms uncovered that the most of *P*. *cruentum* unigenes belonged to integral component of membrane (38%) (GO:0016021), followed by cytoplasmic part (36%) (GO:0044444) and intracellular organelle (34%) (GO:0043229). Anion binding (31%) (GO:0043168), nucleotide binding (30%) (GO:0000166), nucleoside phosphate binding (30%) (GO:1901265) and ribonucleotide binding (27%) (GO:0032553) dominated the molecular function terms. Macromolecule metabolic process (33%) (GO:0043170), cellular nitrogen compound metabolic process (29%) (GO:0034641) and organonitrogen compound metabolic process (26%) (GO:1901564) were the most represented categories in BP terms (**Fig 3A**).

Pathway analysis can ensure to acquire a comprehensive understanding of the biological significance of these unigenes in *P*. *cruentum*. By KEGG analysis, functional pathway annotation uncovered that the most overrepresented pathways were translation, carbohydrate metabolism, folding, sorting, degradation, amino acid metabolism, and energy metabolism, followed by metabolism of cofactors and vitamins, environmental adaptation, nucleotide metabolism, and lipid metabolism (**Fig 3B**).

### Identification of DEGs under nitrogen starvation in *P*. *cruentum*

**Fig 4:**
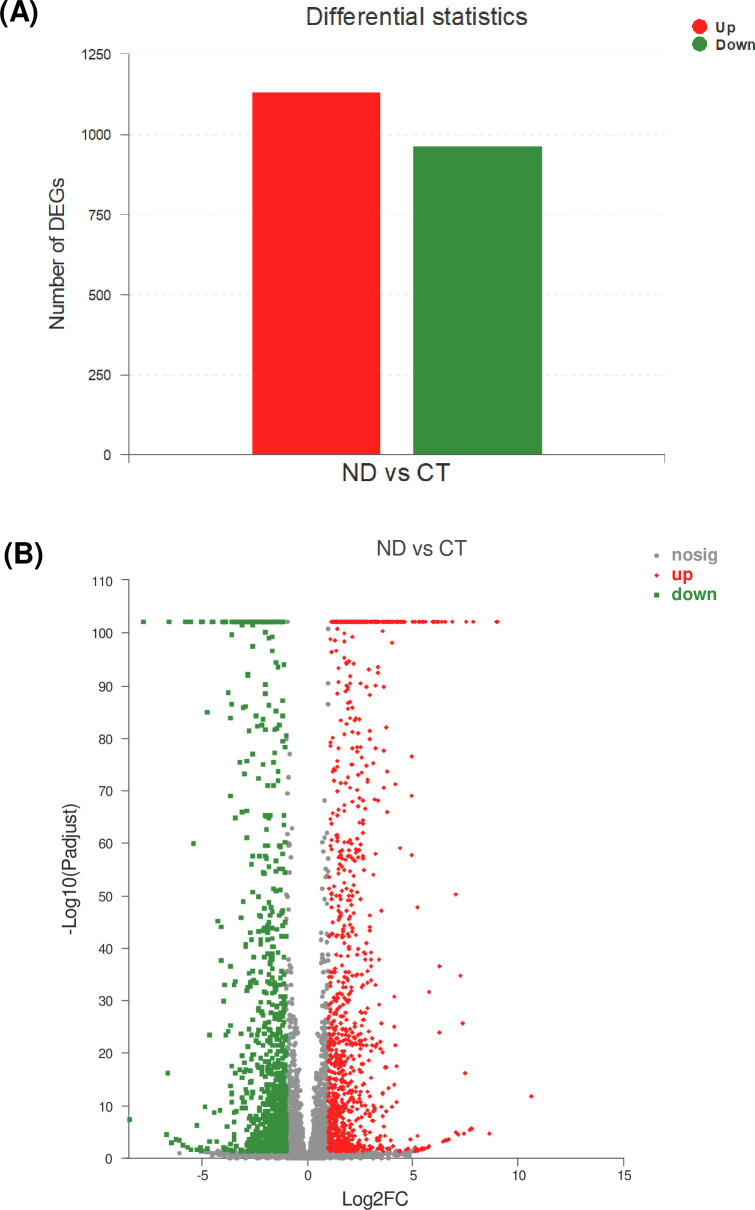
Differential expression unigenes in *P*. *cruentum* of two different cultivation conditions. **(A)** The number of differential expression genes (DEGs) under ND (nitrogen depletion) vs CT (nitrogen repletion). At least 2-fold down-regulation or up-regulation was calculated under nitrogen starvation condition. **(B)** Volcano plot of the differential gene expression patterns between CT and ND. Reds pots represent up-regulated DEGs, green spots represent down-regulated DEGs, and gray spots represent unigenes that did not change significantly under nitrogen deprivation (ND) treatment.

To further investigate the molecular mechanism under nitrogen deprivation, the differential expression unigenes (DEGs) in ND (nitrogen depletion for 72 h) compared to control (CT; nitrogen repletion for 0 h) were detected. As a result, there were 2,100 DEGs (fold change ≥ 2; *p*-value < 0.05) identified in total (**Fig 4A**), including 1,134 upregulated and 966 downregulated unigenes (**Fig 4B and S1 Dataset**). The average level of the RNA expression under two different conditions was also analyzed (**S5 Fig**).

**Fig 5:**
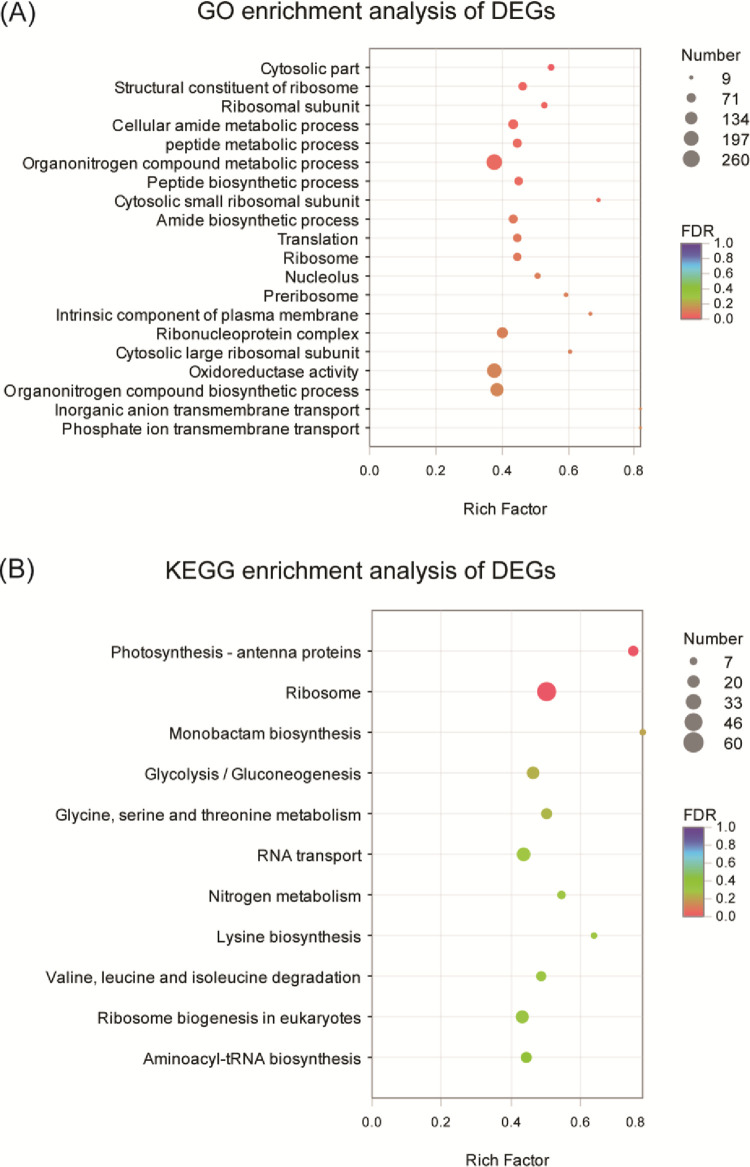
Functional enrichment analysis of differential expression unigenes under ND in *P*. *cruentum*. (**A**) Functional enrichment of differential expression unigenes (DEGs). By Gene ontology (GO) analysis, the DEGs were systematically classified into three subcategories, including biological process (BP), cellular component (CC), and molecular function (MF). Each bar represents the number of unigenes under each subcategory. (**B**) Enrichment analysis of differential expression genes (DEGs) by KEGG. Five main KEGG metabolism pathway categories are carbon/nitrogen and lipid metabolisms, environmental adaptation processing, genetic information processing, cellular processes, and organismal systems. Each bar represents the numbers of unigenes classified under each KEGG term. The vertical and horizontal axises represent different pathways and rich factor, respectively. The color shades correspond to different q-value and the size of the dot represents the number of DEGs.

To survey the function of DEGs, we performed GO functional enrichment analysis. The result showed that organonitrogen compound metabolic process, cellular nitrogen compound biosynthetic process, and organonitrogen compound biosynthetic process were significantly enriched (corrected *P*-value < 0.05) after exposure to ND condition (**Fig 5A**). The processes including RNA binding, ribonucleoprotein complex, and were also affected by ND. Moreover, it is worth noting that all these terms remarkedly showed the trend of downregulation, which indicated these molecular functions were inhibited under ND. In addition, oxidoreductase activity displayed a differential regulation under ND, which implies that nitrogen deprivation would alter homeostasis of metabolism. Other processes involved in membrane transport were also affected by ND, including intracellular non-membrane-bounded organelle and non-membrane-bounded organelle (**Fig 5A and S1 Dataset**). Moreover, some membrane transports potentially participated in organelle (chloroplast and mitochondria) embraced photosynthetic membrane, thylakoid membrane, chloroplast part and inorganic anion transmembrane transport (**S1 Dataset**).

To investigate the pathways influenced by ND, DEGs were further mapped to the KEGG database. Similar to GO functional enrich analysis, the result showed that ribiosome, photosynthesis, and nitrogen metabolism were significantly enriched (**Fig 5B**). Taken together, ten up- (or down-) regulated significantly enriched terms including photosynthesis-antenna proteins, ribosome, monobactam biosynthesis, glycolysis/gluconeogenesis pathways, glycine, serine and threonine metabolism, nitrogen assimilation pathways, lipoic acid metabolism, and glycerophospholipid metabolism (**Fig 5B and S1 Dataset**).

### Nitrogen metabolism affected by nitrogen starvation in *P*. *cruentum*

**Fig 6:**
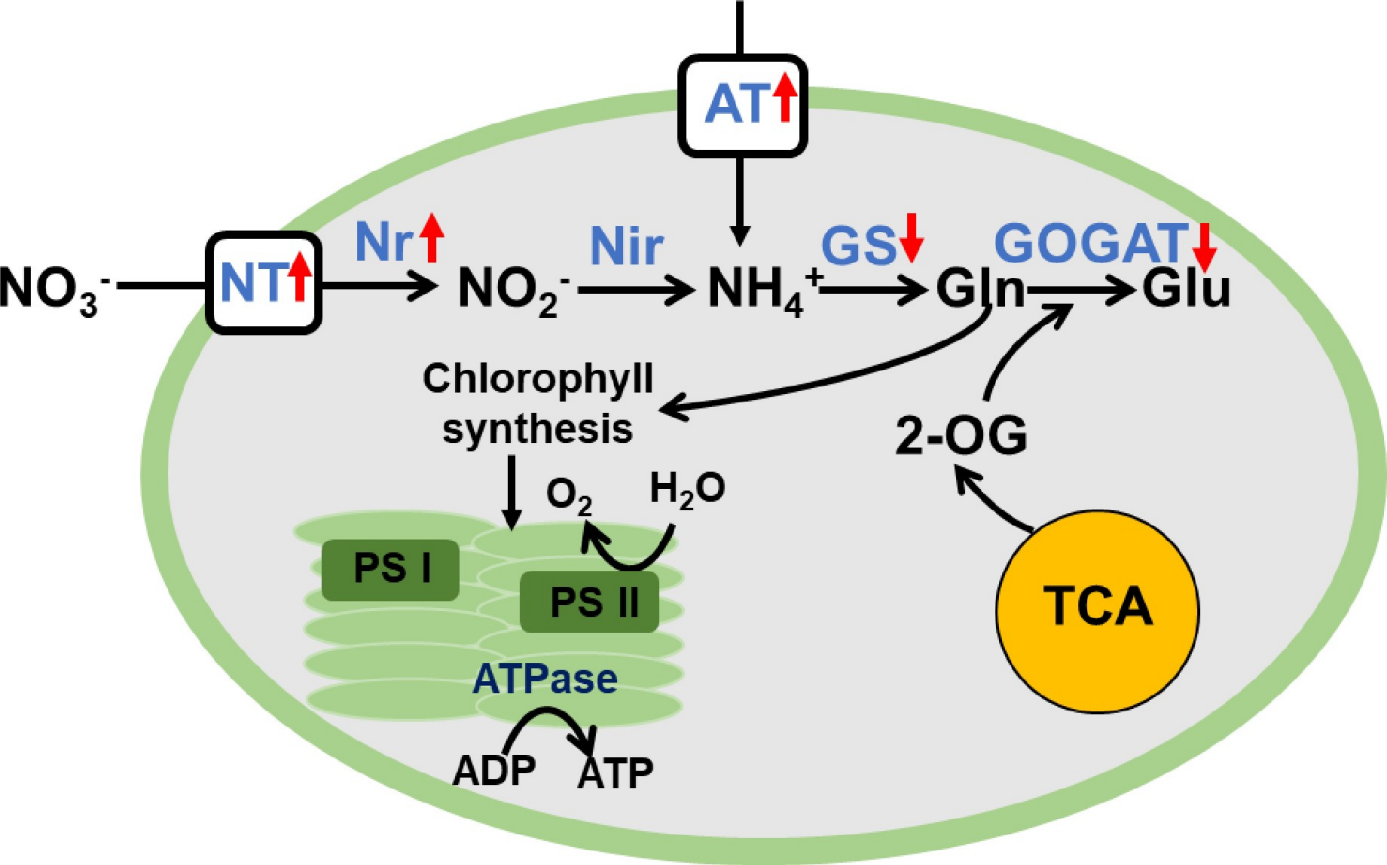
Differential expression analysis of genes related to nitrogen metabolism under ND in *P*. *cruentum*. The pathway of nitrogen assimilation was briefly constructed and the transcriptional change of sever unigenes was presented with arrow. “↑” and “↓” represented upregulation and downregulation under ND, respectively.

To investigate the effect of nitrogen deprivation on the nitrogen metabolism, primary pathways related to nitrogen assimilation was analyzed. As for nitrate assimilation, in *P*. *cruentum* transcriptome, three homologies of nitrate reductase were annotated, which is responsible for reducing nitrate to ammonium into amino acid synthesis. Among them, under ND vs CT, two unigenes (TRINITY_DN20_c0_g1 and TRINITY_DN20_c0_g2) coding nitrate reductase displayed 493- and 13-fold upregulation, respectively (**Fig 6 and S1 Dataset**). Notably, two unigenes (TRINITY_DN867_c0_g2 and TRINITY_DN867_c0_g1) encoding nitrate transporter were also upregulated 93- and 4.4- fold (**Fig 6**). This result demonstrated that nitrate assimilation was activated under ND, which is consistent with that in majority of microalgae [22]. Additionally, one unigene coding ammonium transporter (TRINITY_DN5475_c0_g1) was upregulated by 8.1-fold. By contrast, ND can improve nitrogen transport and stimulate reduction processes. Albeit nitrite assimilation was also essential during nitrate metabolism, the unigenes related to nitrite reductase (responsible for reducing nitrite to ammonium) weren’t annotated. Therefore, we can’t observe whether they were regulated by ND.

In yeast and fungi, to cope with nitrogen deprivation, they often employ the most common strategy that to maintain the level of nitrogen inside the cell by up-regulation of nitrogen transporters, or otherwise the cells might undergo morphological modifications like generation of pseudohypha to combat the nitrogen stress [23]. In plants, under nitrogen stress, nitrate or nitrite reductase was also activated in Arabidopsis [24]. In *Chlamydomonas* [25] and diatom *P*. *tricornutum* [26], nitrate reductase increased 10- and 4-fold under nitrogen stress, respectively. Moreover, the knockdown of nitrate reductase would lead to 43% increase in cellular lipid content in *P*. *tricornutum*. Therefore, the pathway of nitrate assimilation might be important target for improving lipid production.

In addition, the glutamine synthetase-glutamate synthase (GS-GOGAT) cycle can assimilate intracellular ammonium from nitrate metabolism (**Fig 6**), which is present in plant and microalgae for nitrogen assimilation. The GS-GOGAT cycle provides an entry route for reduced inorganic nitrogen into all organic nitrogenous compounds in plants [27]. In *P*. *cruentum* transcriptome, TRINITY_DN1447_c0_g1 unigenes coding glutamine:2-oxoglutarate amidotransferase (GOGAT) and TRINITY_DN294_c0_g1 encoding glutamate synthase (GS) were observed. Both are involved in GS/GOGAT cycle that incorporates ammonium into amino acids (**Fig 6**) [18]. Under ND, the transcript abundance of GS (TRINITY_DN294_c0_g1) displayed a 3.8-fold decrease in response to ND (**Fig 6 and S1 Dataset**). In *Chlamydomonas*, partial gene transcripts or proteins increased under nitrogen limitation at the transcript or protein level [25]. In diatom, most ammonium from nitrate or urea assimilation was assimilated in the organelle-specific GS-GOGAT cycles with only modest levels of ammonium flux into the urea cycle simulated on either nitrogen source [28]. Therefore, the GS-GOGAT cycle is also essential for nitrogen metabolism in both green and red microalgae.

### Change of carbon fixation and central carbon metabolism in response to nitrogen starvation

To interrogate the effect of nitrogen starvation on the central carbon metabolism and photosynthetic carbon fixation in *P*. *cruentum*, we firstly surveyed the transcript changes of these pathways including carbon concentration mechanism (CCM), Calvin-Benson cycle, glycolysis and gluconeogenesis. In *P*. *cruentum* transcriptome, a total of five carbonic anhydrases (CA: responsible for the inter-conversion between CO_2_ and HCO_3_^-^) unigenes as key components of CCM were annotated. Among them, three CA unigenes showed a downregulation under ND condition, including TRINITY_DN5668_c0_g1 (beta-type), TRINITY_DN1722_c0_g1 (beta-type) and TRINITY_DN3714_c0_g1 (gamma-type). Interestingly, they were downregulated by 13.8-, 5.2- and 2.7-fold (**Table 3**), respectively, which implies that CCM was affected by ND. The gamma-type CA (TRINITY_DN3714_c0_g1) was predicted to target to mitochondrion and other two beta-type CAs could localize to chloroplast, which suggest ND affected carbon concentration mechanisms. In microalga *Chlamydomonas* [25] and *Nannochloropsis oceanica* [8], CAs were regulated at the RNA level by nitrogen stress. Unfortunately, we didn’t annotate the candidate unigenes (such as bicarbonate transporter; another key component of CCM) responsible for bicarbonate transport. However, sodium hydrogen exchanger (TRINITY_DN2419_c0_g1) and BASS family transporter: sodium ion/bile acid (TRINITY_DN2381_c0_g2) might be involved in bicarbonate transport and showed a 5.3- and 2.4-fold upregulation respectively (**Table 3**), because they were experimentally demonstrated as potential bicarbonate transporter proteins in diatom [29]. Thus, ND interrupted CCM in *P*. *cruentum* based on the transcriptional change of CA and bicarbonate transporter.

**Table 3:** Differential gene expression participated in carbon metabolism in *P*. *cruentum*.

Gene ID	Gene name	Abbreviation	Fold change (ND Vs CT; fold)
TRINITY_DN5668_c0_g1	Carbonic anhydrase	CA	↓ 13.8
TRINITY_DN1722_c0_g1	Carbonic anhydrase	CA	↓ 5.2
TRINITY_DN3714_c0_g1	Carbonic anhydrase	CA	↓ 2.7
TRINITY_DN2419_c0_g1	sodium hydrogen exchanger	SHE	↑ 5.3
TRINITY_DN2381_c0_g2	BASS family transporter	BT	↑ 2.4
TRINITY_DN3833_c0_g1	sedoheptulose-1,7-bisphosphatase	SB	↓ 2.4
TRINITY_DN5219_c0_g1	phosphoribulokinase	PB	↓ 4.9
TRINITY_DN3773_c0_g1	Fructose-1,6-bisphosphatase	FB	−
TRINITY_DN1303_c0_g1	phosphoglycerate kinase	PGK	↓ 3.1
TRINITY_DN6107_c0_g1	glyceraldehyde-3-phosphate dehydrogenase	GPD	↓ 9.1
TRINITY_DN2702_c0_g1	triosephosphate isomerase	TI	−
TRINITY_DN5407_c0_g1	Pyruvate kinase	PK	↑ 5.3
TRINITY_DN2244_c0_g1	glycosyl hydrolase	GH	↓ 2.2

“↓” and “↑” represented up- and down-regulation respectively and “−” was remarked as no change.

Secondly, in terms of photosynthetic carbon fixation, we particularly focused on the unigenes participated in Calvin cycle. As for two subunits of ribulose-1,5 bisphosphate carboxylase/oxygenases related to the Calvin-Benson cycle, they didn’t show significantly differential expression in the transcriptome of *P*. *cruentum*. Some other unigenes related to Calvin cycle were annotated in the transcriptome of *P*. *cruentum*, including chloroplastic sedoheptulose-1,7-bisphosphatase (TRINITY_DN3833_c0_g1), phosphoribulokinase (TRINITY_DN5219_c0_g1), Fructose-1,6-bisphosphatase (TRINITY_DN3773_c0_g1), phosphoglycerate kinase (TRINITY_DN1303_c0_g1), glyceraldehyde-3-phosphate dehydrogenase (TRINITY_DN6107_c0_g1), and triosephosphate isomerase (TRINITY_DN2702_c0_g1). Among them, phosphoribulokinase (TRINITY_DN5219_c0_g1), Phosphoglycerate kinase (TRINITY_DN1303_c0_g1), and glyceraldehyde-3-phosphate dehydrogenase (TRINITY_DN6107_c0_g1) displayed a 4-, 2-, and 9- fold downregulation, respectively (**Table 3 and S1 Dataset**). Moreover, we didn’t observe that other genes of Calvin cycle were upregulated. This means that the function of Calvin cycle was reduced under ND in *P*. *cruentum*, which will result in the accumulation of cycle metabolic intermediates. Intriguingly, those genes of Calvin cycle were upregulated under nitrogen stress in green algal *Chlorella sorokiniana* [5, 30]. Therefore, red algal *P*. *cruentum* might employ different mechanism to cope with nitrogen stress.

Thirdly, as for glycolysis and gluconeogenesis metabolic pathways, transcripts of several unigenes related to glycolytic pathways, like pyruvate kinase (PK) and glycosyl hydrolase, showed differential regulation under ND. Pyruvate kinase (TRINITY_DN5407_c0_g1), an allosteric enzyme responsible for converting phosphoenol pyruvate to pyruvate in glucose metabolism, displayed a 7.9-fold upregulation. However, glycosyl hydrolase (TRINITY_DN2244_c0_g1) was downregulated by 2.2-fold at the RNA level (**Table 3 and S1 Dataset**). On the contrary, no drastic changes were observed in pyruvate kinase expression after N starvation in the green alga *Chlamydomonas reinhardtii* [31]. Hence, single red microalgae adopted to different adaption mechanisms.

### Photosynthesis affected in response to nitrogen starvation

To survey the effect of ND on photosynthesis, transcripts of the unigenes related to photosystem, light harvesting system and chlorophyll biosynthesis were interrogated in *P*. *cruentum*. We observed that a majority of unigenes participating in light harvesting proteins and photosystem displayed downregulation under ND. For instance, light-harvesting of photosystem I (TRINITY_DN1494_c0_g1), light harvesting chlorophyll a b-binding protein (TRINITY_DN4878_c0_g1 and TRINITY_DN5172_c0_g1), and chloroplast light-harvesting complex I protein precursor Lhca4 (TRINITY_DN1665_c0_g1) were downregulated by 22.7-, 6.4-, and 35.2-fold (**Table 4 and S1 Dataset**). In addition, the transcriptional abundance of Photosystem II repair PSB27- chloroplastic (TRINITY_DN676_c0_g1) and apoprotein A1 of photosystem I (TRINITY_DN3937_c0_g1) were also decreased 8.8- and 33.3-fold, respectively (**Table 4 and S1 Dataset**). However, the unigene of D1 reaction center protein of photosystem II (TRINITY_DN3429_c0_g1) was up-regulated by 170-fold. Overall, the transcript levels of a majority of the unigenes related to photosynthetic electron transport and photophosphorylation was reduced under ND in *P*. *cruentum*. This observation is consistent with the content of *Chla*, which suggest that its photosynthesis would be impaired. These results are consistent with observation occurred in a majority of microalgae such as *Chlamydomonas* [25], *Nannochloropsis* [32], and diatom *P*. *tricornutum* [6, 33]. In *Chlamydomonas*, at the RNA level, those genes encoding the photosynthetic light harvesting complexes were strongly decreased under nitrogen deficiency cells [25]. In *Nannochloropsis*, the capacity of photosynthesis was reduced under nitrogen deprivation [32]. *P*. *tricornutum*, photosynthesis was also decreased under nitrogen or phosphorus deficiency [6, 33]. Taken together, photosynthesis is closely linked to whole physiology and energy conversion, in this study, most of the transcript abundance of unigenes involved in photosynthesis were downregulated during nitrogen starvation in *P*. *cruentum*. These results indicated that energy conversion would be retarded and the growth would be also influenced.

**Table 4:** Differential gene expression related to photosynthesis in *P*. *cruentum*.

Gene ID	Gene name	Abbreviation	Fold change (ND Vs CT; fold)
TRINITY_DN1494_c0_g1	light-harvesting of photosystem I	LHC	↓ 22.7
TRINITY_DN4878_c0_g1	light harvesting chlorophyll a b-binding protein	LHC	↓ 6.4
TRINITY_DN676_c0_g1	Photosystem II repair PSB27	PSB	↓ 8.8
TRINITY_DN3937_c0_g1	Protein A1 of photosystem I	A1	↓ 33.3
TRINITY_DN3429_c0_g1	D1 reaction center protein of photosystem II	D1	↑ 170.1
TRINITY_DN5172_c0_g1	Light harvesting chlorophyll a b-binding protein	LHC	↓ 35.3

“↓” and “↑” represented up- and down-regulation respectively and “−” was remarked as no change.

### Lipid metabolism affected by nitrogen starvation

**Fig 7:**
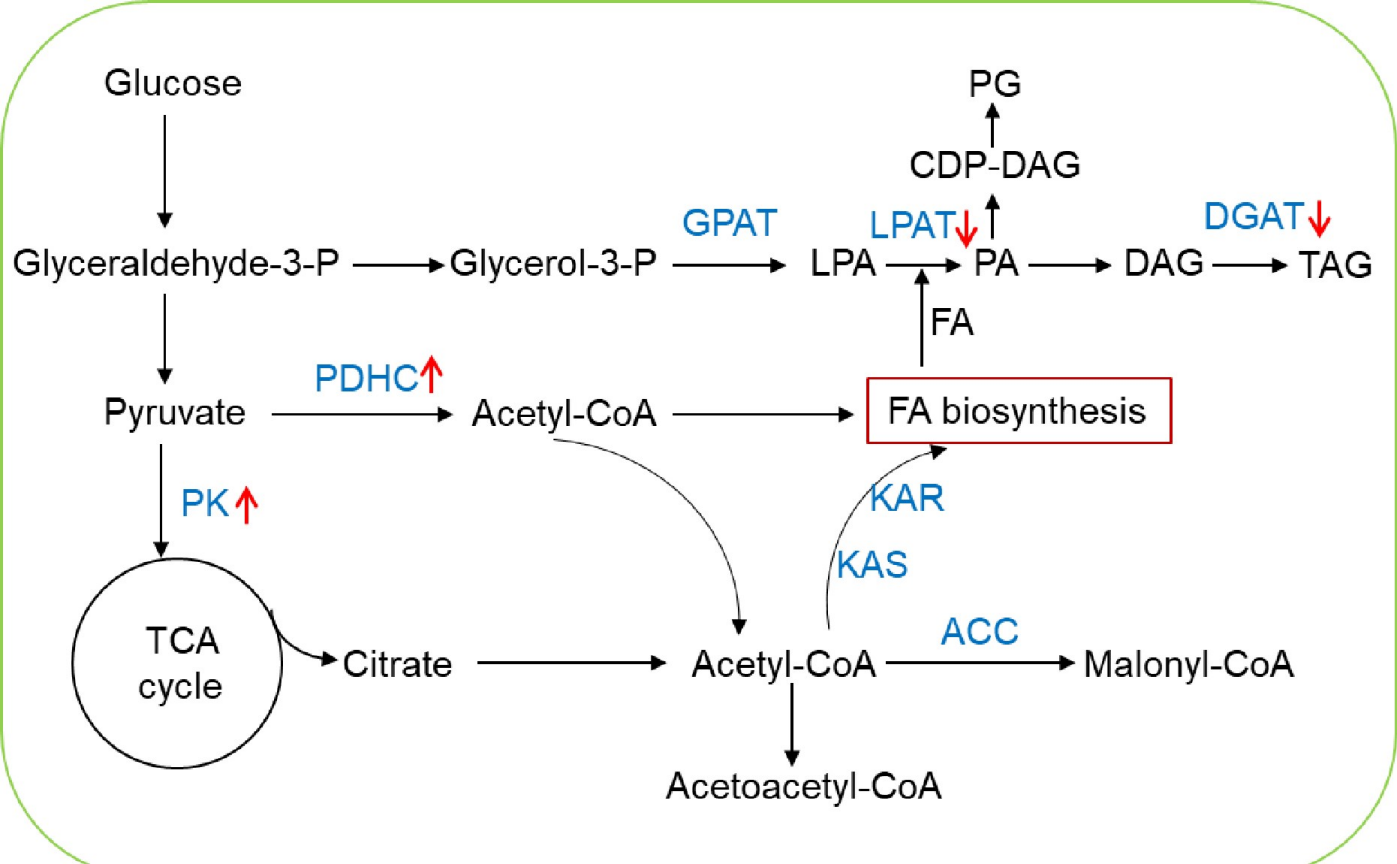
Differential expression analysis of genes related to lipid metabolism under ND in *P*. *cruentum*. The pathway of lipid metabolism was briefly constructed and the transcriptional change of sever unigenes was presented with arrow of “↑” and “↓” represented upregulation and downregulation under ND, respectively.

Lipid metabolism was largely regulated by nitrogen stress in high plants and algae [3], thus we analyzed its regulation of fatty acid biosynthesis and TAG in *P*. *cruentum*. As for fatty acid biosynthesis, several key enzymes showed no differential transcriptional regulation in *P*. *cruentum*. For instance, the transcripts of acetyl-CoA carboxylase (TRINITY_DN1906_c0_g1), 3-hydroxyacyl-ACP dehydrase (TRINITY_DN796_c0_g1), and ketoacyl-ACP reductase (TRINITY_DN1026_c0_g1) were not regulated by ND. However, the transcript abundances of 3-ketoacyl-acyl carrier protein synthase (TRINITY_DN2527_c0_g1) and enoyl-ACP reductase (TRINITY_DN1209_c0_g1) were downregulated by 2.2- and 3.2-fold, respectively (**S1 Dataset**). Notably, Pyruvate dehydrogenase complex (TRINITY_DN606_c0_g1) displayed 3.1-fold upregulation (**Fig 7 and S1 Dataset**). Additionally, Pyruvate decarboxylase (PDC; TRINITY_DN5826_c0_g1) showed 4.1-fold upregulation, which is responsible for the conversion of pyruvate to acetyl coenzyme A as E1 component of the pyruvate dehydrogenase (PDH) complex in *P*. *cruentum*. The change of these transcripts might be related to the change of LC-PUFAs (LA, ARA and EPA). In *Chlamydomonas*, all enzymes related to fatty acid biosynthesis were reduced at the transcriptional level under nitrogen deprivation [25]. In *Nannochloropsis*, most of the genes involved in a type II FAS-based de novo FA biosynthesis in the plastid were downregulated [8]. However, red microalga *P*. *cruentum* presented different expression pattern, which suggests that *P*. *cruentum* would employ different strategy to cope with nitrogen stress.

As for TAG synthesis, we focused on several key step enzymes for TAG synthesis. For instance, lysophosphatidic acid acyltransferase (LPAT; TRINITY_DN3436_c0_g1), a key enzyme involved in catalyzing fatty acid chains into 3-phosphoglycerate and promoting further production of oil in the form of TAG in the Kennedy pathway, displayed an 8.3-fold downregulation under ND in *P*. *cruentum* (**Fig 7 and S1 Dataset**). The transcript abundance of diacylglycerol acyltransferase 1 (DGAT1; TRINITY_DN756_c0_g2) also showed a 10-fold downregulation, which catalyzes the final and committed step in the Kennedy pathway for TAG biosynthesis (**Fig 7 and S1 Dataset**). However, we didn’t observe the increased or decreased transcripts of other homologys of LPAT or DGAT. Distinctively, in *Chlamydomonas*, those genes encoding the TAG biosynthesis-specific acyltransferases and the glycerol-3-phosphate dehydrogenase isozymes displayed a significant upregulation at the mRNA level [34]. In *Nannochloropsis*, the upregulations of some genes such as diacylglycerol acyltransferase (DGAT), lipase, and lipid body associated proteins were also observed [8, 35–37]. In addition, the transcript of acetoacetyl-CoA thiolase (also called thiolase II; TRINITY_DN2946_c0_g1) responsible for the thiolysis of acetoacetyl-CoA was downregulated, which involved in biosynthetic pathways such as beta-hydroxybutyric acid synthesis or steroid biogenesis. However, single-cell red *P*. *cruentum* presented different expression pattern compared to other green microalgae, which suggests that *P*. *cruentum* would lanuch different mechanisms to reply to nitrogen stress.

## Conclusion

This study not only firstly provided *de novo* transcriptome assembly datasets of *P*. *cruentum*, but also new biological insights into the mRNA transcripts of genes associated with photosynthesis, carbon/nitrogen metabolism, fatty acid biosynthesis and lipid metabolism in response to nitrogen deprivation. Based on this study, it is clear that this observation can contribute to the understanding of metabolic reprogramming under ND and the regulation of metabolites for both fundamental and applied research. Moreover, the *P*. *cruentum*’s transcriptome data could be a contribution for analyzing the physiology, elucidating its evolution of the Rhodophyta, and designing metabolic pathway of synthetic biology.

## Materials and methods

### Culture conditions of *Porphyridium cruentum*

*Porphyridium cruentum* GY-H56 from Shanghai Guangyu Biological Technology Co., Ltd was inoculated into the modified f/2 liquid medium, which was prepared with 35 g L^-1^ sea salt (Real Ocean, USA), 1 g L^-1^ NaNO_3_, 3.65 mg L^-1^ FeCl_3_*6H_2_O, 67 mg L^-1^ NaH_2_PO_4_*H_2_O, 4.37 mg L^-1^ Na_2_EDTA*2H_2_O, trace metal mix (0.36 mg L^-1^ MnCl_2_*4H_2_O, 0.0126 mg L^-1^ NaMoO_4_*2H_2_O, 0.0196 mg L^-1^, CuSO_4_*5H_2_O, 0.044 mg L^-1^ ZnSO_4_*7H_2_O, and 0.01mg L^-1^ CoCl_2_), and vitamin mix (2.5 μg L^-1^ biotin, 2.5 μg L^-1^ VB_12_, and 0.5 μg L^-1^ thiamine HCl) [38]. The cells were cultured in f/2 medium at 25°C with continuous irradiation (light intensity: 50±5 μmol m^-2^ s^-1^) in a 1 L column reactor (inner diameter: 5 cm). The seed cultures were bubbled with air aeration (no additional CO_2_ added; aeration rate: 0.5 vvm) under phototrophic mode. At the logarithmic phase (OD_750_ = 3.0), microalgal cells were harvested by 2,500 g centrifugation, one was sampled for RNA extraction as control (CT) with three replicates and the other was washed with fresh medium (nitrogen-free) for three times before being used for the following experiments. Cell pellets were inoculated into Nitrogen-free f/2 medium. Cultures started with the same initial cell concentration of OD_750_ = 3.0 and were exposed to continuous illumination with same light intensity. Cell aliquots were sampled for RNA isolation after being transferred to the nitrogen-free conditions at 72 h as nitrogen-deprivation samples (ND) with three replicates.

### Measurement of lipid, carbohydrate, protein and *Chla*

To quantify the amount of carbohydrate, protein and lipid, *P*. *cruentum* cells were harvested after 24h cultivation by centrifugation (at 4000 rpm for 5 min) under CT and ND conditions. To dried algal powder, cells were lyophilized for two days. Extraction and assaying of lipid, carbohydrate and protein in the microalgal biomass were performed based on previous published protocols [39].

*Chla* content was measured using a modified extraction method [40]. Briefly, a sample of the algal culture was centrifuged at 2,500 × g, and the filter was extracted in the dark for 24 h in 100% ethanol saturated with MgCO_3_. The samples were then centrifuged and the supernatant was analyzed spectrophotometrically. Extinction values at 632, 649, 665 and 750 nm were recorded using a UV-Vis spectrophotometer. For PE extraction, a sample of 2 mL from each cell culture was centrifuged at 4500 g for 15 min and the supernatant was discarded. The sediment was mixed with 2 mL of distilled water and was vortexed until homogenization. The suspension was frozen at -20°C for one hour, and then it was thawed and sonicated in an ice bath for 10 min. The sample was vortexed until homogenization and repeat steps until completing five cycles. After that, the samples were centrifuged at 4500 rpm for 15 min. The pink supernatant was recovered and measured in a spectrophotometer at 564, 592 and 455 nm. The obtained data were substituted in the Beer and Eshel equation to calculate the concentration of PE:

PE (mg mL^−1^) = [(OD564nm−OD592nm)−(OD455nm−OD592nm)0.2]×0.12.

### Analysis of lipids

Lipid extraction for fatty acid analysis was conducted following the method of Jia [39]. In brief, about 0.1 g of lyophilized biomass of each sample was extracted with a chloroform–methanol–water solution. The lipid-containing chloroform phase in the substratum was dried to powder under a nitrogen stream. Fatty acid methyl esters (FAMEs) were prepared by esterification of the powdered lipids in a KOH-methanol solution with the C17:0 ester containing cyclohexane as the internal standard. The upper layer of the mixture was separated for FAME composition analysis. The FAME composition analysis was conducted with a GC-MS system (an Agilent 6890 GC fitted with an Agilent 5975 MSD). The injection volume was 1 μL for each sample. The temperature of the injector and detector, the column flow rate, and the split ratio were 250°C, 1.2 mL min^−1^, and 1:50, respectively. The running temperature was set as follows: 40°C for 1 min, heating to 230°C at 20°C min^−1^, held at 230°C for 1 min, heating to 270°C at 3°C min^−1^, and held at 270°C for 2 min. An internal standard was used to quantify the weight (mg) of each fatty acid, and its cellular content (mg g^−1^) was calculated in terms of its weight per gram of microalgal powder.

### Total RNA extraction

Total RNA was isolated and extracted from the microalgal cells according to the previous Li’s protocols (Invitrogen, Carlsbard, CA, USA) and genomic DNA was erased using DNase I (TaKara) [8]. Briefly, the cells were harvested by centrifugation for 5 min at 2,500 g, and then were immediately quenched with liquid N_2_ and stored in -80°C freezer. The cell pellets were subsequently ground under liquid nitrogen using a mortar and pestle and RNA was extracted using TRIzol® Reagent and chloroform. RNA extract was treated with DNase I at 37°C for 30 min, and then mixed thoroughly with equal volume of phenol:chloroform (1:1). After centrifuging (10,000 g, for 10 min at 4°C), 0.75 mL of 8 M LiCl was added for precipitation. Then the integrity and purity of the total RNA quality was assessed by 2100 Bioanalyser (Agilent Technologies, Inc., Santa Clara CA, USA) and quantified using the ND-2000 (NanoDrop Thermo Scientific,Wilmington, DE, USA) and Qubit 3.0 (Life Technologies, Life Technologies). Only high-quality RNA sample (OD260/280 = 1.8~2.2 and OD260/230≥2.0) was used to construct sequencing library.

### mRNA sequencing library preparation and Illumina Novaseq 6000 sequencing

RNA purification, reverse transcription, library construction and sequencing were performed at Shanghai Majorbio Bio-pharm Biotechnology Co., Ltd. (Shanghai, China) according to the manufacturer’s instructions (Illumina, San Diego, CA). These four mRNA-seq libraries were constructed with Illumina TruSeq^TM^ RNA sample preparation Kit (San Diego, CA). Total RNA was purified and isolated by oligo-dT-attached magnetic beads, and then RNA was fragmented by fragmentation buffer. Next, these short RNA fragments were taken as templates to synthesize double-stranded cDNA using a SuperScript double-stranded cDNA synthesis kit (Invitrogen, CA) with random hexamer primers. Then the synthesized double-stranded cDNA was subsequently subjected to end-repair, phosphorylation and ‘A’ base addition according to Illumina’s library construction protocol. As for selecting cDNA target fragments of 200–300 bp, libraries were run on 2% Low Range Ultra Agarose after PCR-amplification by Phusion DNA polymerase (New England Biolabs, Boston, MA) for fifteen cycles. After quantification, four RNA sequencing libraries were performed to sequence in single lane on an Illumina Novaseq 6000 platform (Illumina, San Diego, CA) for 2×150bp paired-end reads.

### *De novo* transcriptome assembly and functional annotation

The raw paired-end reads of mRNA-seq were trimmed and data quality was controlled using software of SeqPrep (https://github.com/jstjohn/SeqPrep) and Sickle (https://github.com/najoshi/sickle) with default parameters. Then clean data from the samples (CT) were employed to perform *de novo* assembly with Trinity (https://github.com/trinityrnaseq/trinityrnaseq) [21]. All the assembled transcripts were searched against the protein nonredundant (NR) of NCBI, String, and KEGG databases using BLASTX to identify the proteins that had the highest sequence similarity with the given transcripts to retrieve their function annotations and a typical cut-off E-values less than 1.0×10^−5^ was set. BLAST2GO (http://www.blast2go.com/b2ghome) [41] program was used to get GO annotations of unique assembled transcripts for describing biological processes, molecular functions and cellular components. Metabolic pathway analysis was performed using the KEGG (Kyoto Encyclopedia of Genes and Genomes; http://www.genome.jp/kegg/) [42] (**S6 Fig**).

#### Differential expression analysis and functional enrichment

To identify DEGs (differential expression genes) between two different samples, the expression level of each transcript was analyzed based on the fragments per kilobase of exon per million mapped reads (FRKM) method. To quantify gene and isoform abundances, RSEM (http://deweylab.biostat.wisc.edu/rsem/) [43] was employed. R statistical package software EdgeR (Empirical analysis of Digital Gene Expression in R, http://www.bioconductor.org/packages/2.12/bioc/html/edgeR.html) [44] was utilized for differential expression analysis. In addition, functional-enrichment analysis including GO and KEGG were performed to identify which DEGs were significantly enriched in GO terms and metabolic pathways at Bonferroni-corrected P-value ≤0.05 compared with the whole-transcriptome background. GO functional enrichment and KEGG pathway analysis were performed by two softwares (Goatools (https://github.com/tanghaibao/Goatools) and KOBAS (http://kobas.cbi.pku.edu.cn/home.do) [45]).

### Real-time quantitative PCR

To avoid the bias caused by the different biological replicates, we selected six genes related to lipid accumulation and nitrogen metabolism to perform the RT-qPCR. The same samples with mRNA-seq were used to perform the RT-qPCR analysis. M-MLV reverse transcription kit (Promega, USA) was emplyed to synthesis the cDNA according to the manufacturer’s instruction. Gene specific primers (**S2 Table**) were designed by Vector NTI software. A 20 μL reaction mixture was performed on the real-time PCR LifeCycle480 system (Roche, USA) with the SYBR Green qPCR Kit Master Mix (Roche, USA) according to the manufacturer’s protocol. The cycle threshold value (CT) and differential expression were calculated by the method of 2^-△△CT^ with actin gene of *P*. *cruentum* as the endogenous reference. Each sample was run in triplicate to confirm the reproducibility of the results.

### Statistical analysis

The data were analyzed via one-way ANOVA with a least significant difference test using Excel.

## Supporting information

S1 Fig The length distribution of *P. cruentum* transcriptome assembly sequences. (PDF)

S2 Fig Correlation between two different treatments. (PDF)

S3 Fig qPCR validation of unigenes involved in nitrogen assimilation and lipid metabolisms. Six unigenes related to nitrogen assimilation and lipid metabolisms were used to perform qPCR.
(PDF)

S4 Fig The species distribution of the total homologous sequences in *P. cruentum*. Species distribution of the total homologous sequences was calculated with match (with a cut-off E-value of 1.0E-5) in NR database.
(PDF)

S5 Fig The average level of the RNA expression under two different conditions in *P. cruentum*. Species distribution of the total homologous sequences was calculated with match (with a cut-off E-value of 1.0E-5) in Nr database.
(PDF)

S6 Fig A flow chart of mRNA-seq data analysis. (PDF)

S1 Table De novo assembly length distribution of transcripts in *pone. cruentum*. (DOCX)

S2 Table Primer sequences paired used in real time-qPCR. (DOCX)

S1 Dataset Differential expression genes under two different conditions in *P. cruentum*. (XLSX)

## Abbreviation

PE: phycoerythrin
LC-PUFAs: long-chain polyunsaturated fatty acids
EPA: eicosopentaenoic
ARA: Arachidonic acid
LA: linoleic acid
CT: control
ND: nitrogen depletion
DEGs: differential expression unigenes
GS-GOGAT: glutamine synthetase-glutamate synthase
CCM: carbon concentration mechanism
CA: carbonic anhydrase
PK: pyruvate kinase
DGAT: diacylglycerol acyltransferase
LPAT: lysophosphatidic acid acyltransferase
