# Supplementary figures and images for: Transcriptomic survey reveals multiple adaptation mechanisms in response to nitrogen deprivation in marine Porphyridium cruentum
Source: PLoS One. 2021 Nov 18;16(11):e0259833. doi: 10.1371/journal.pone.0259833 (PMC8601545; doi:10.1371/journal.pone.0259833)

Figure S1

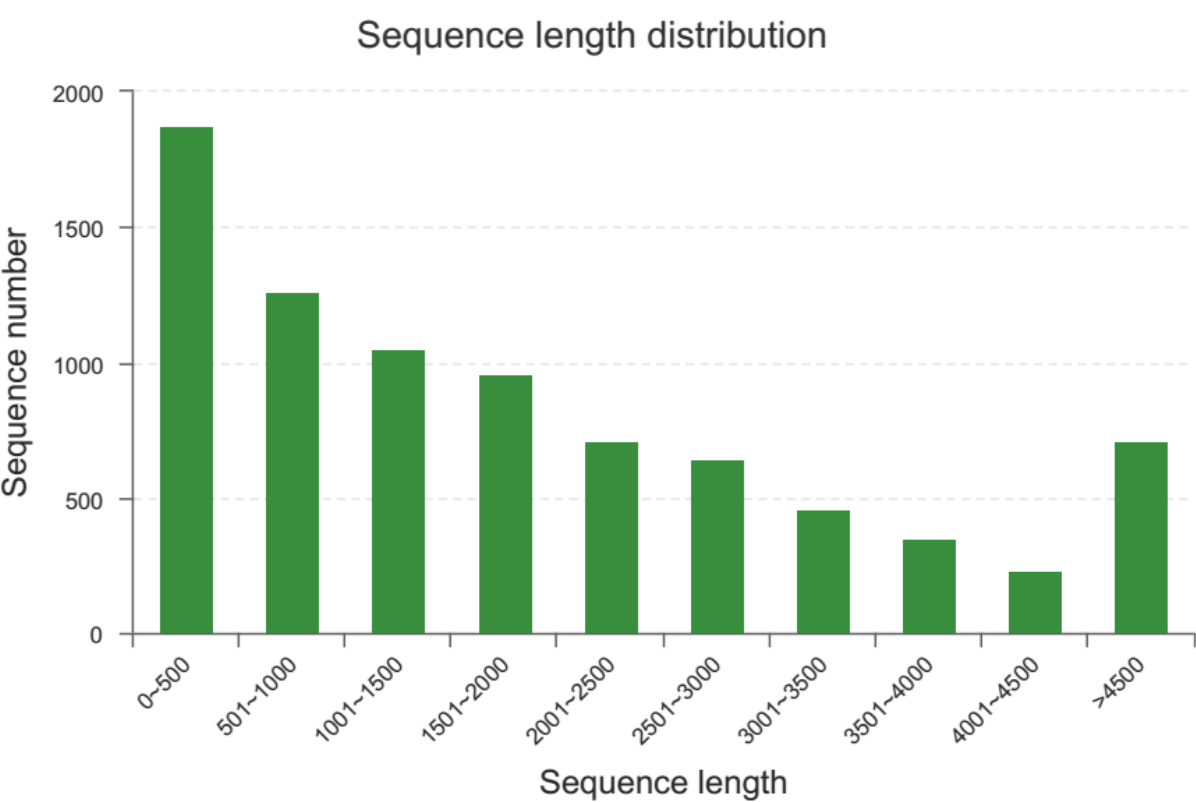

Supplement: S1 Fig — (PDF) [file pone.0259833.s001.pdf]

Figure S2

(A)

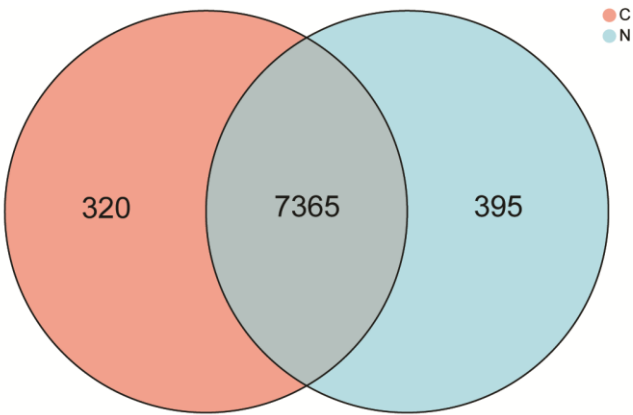

(B)

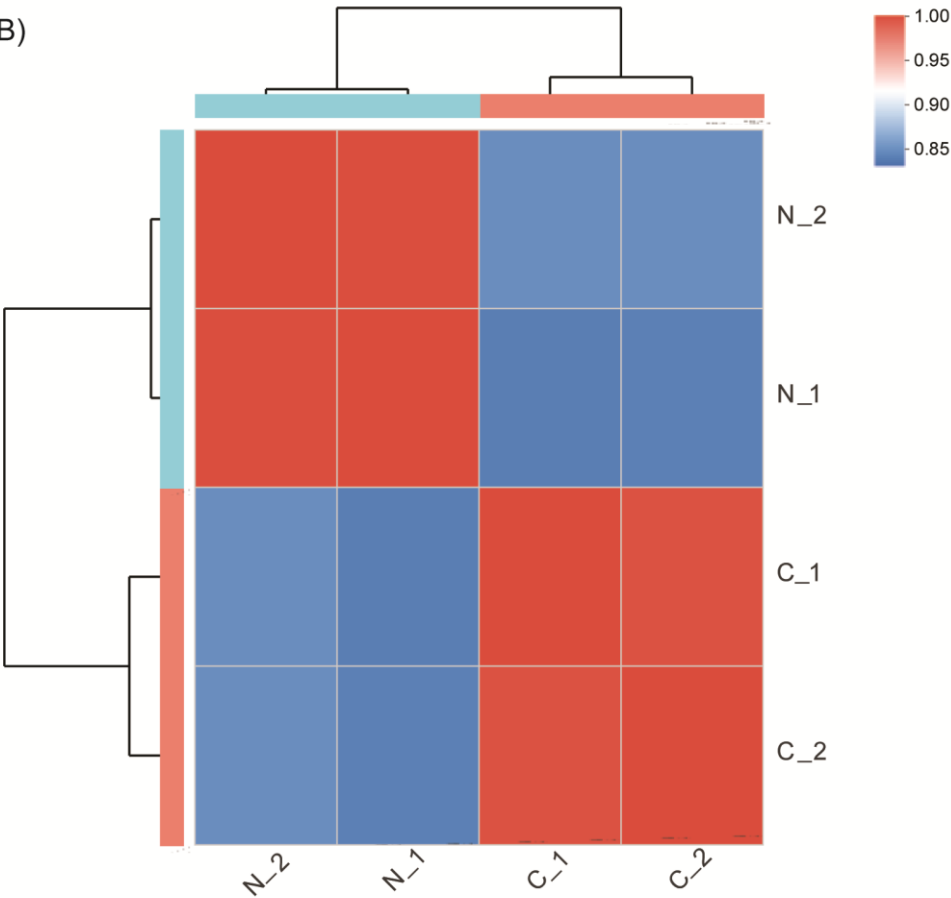

Supplement: S2 Fig — (PDF) [file pone.0259833.s002.pdf]

Figure S3

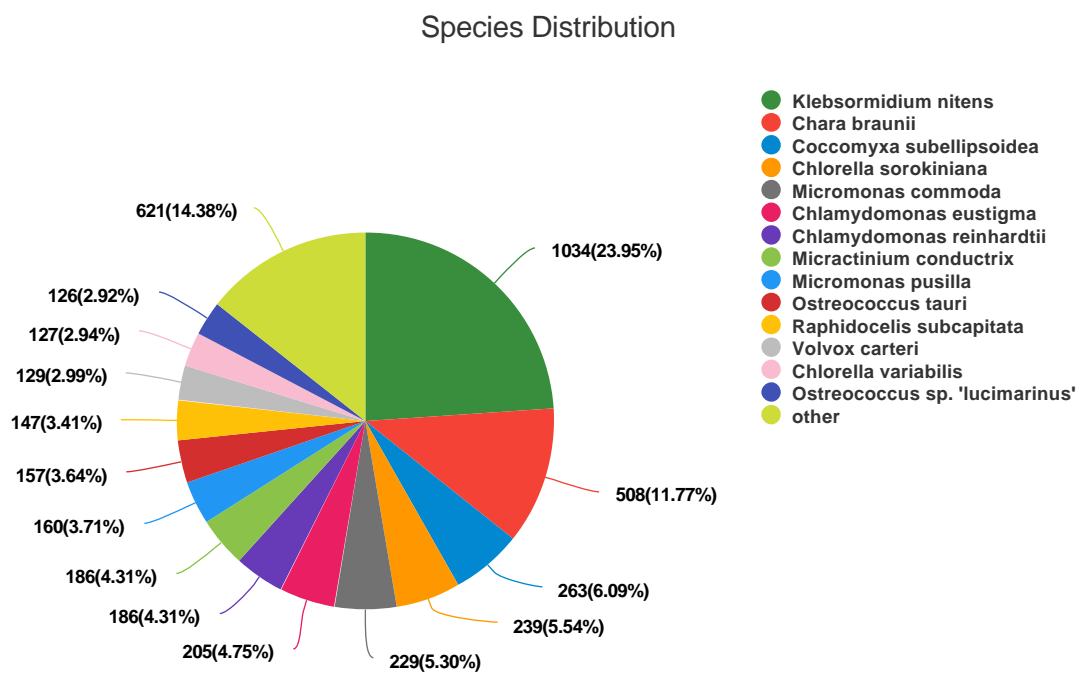

Supplement: S3 Fig — Six unigenes related to nitrogen assimilation and lipid metabolisms were used to perform qPCR. (PDF) [file pone.0259833.s003.pdf]

Figure S4

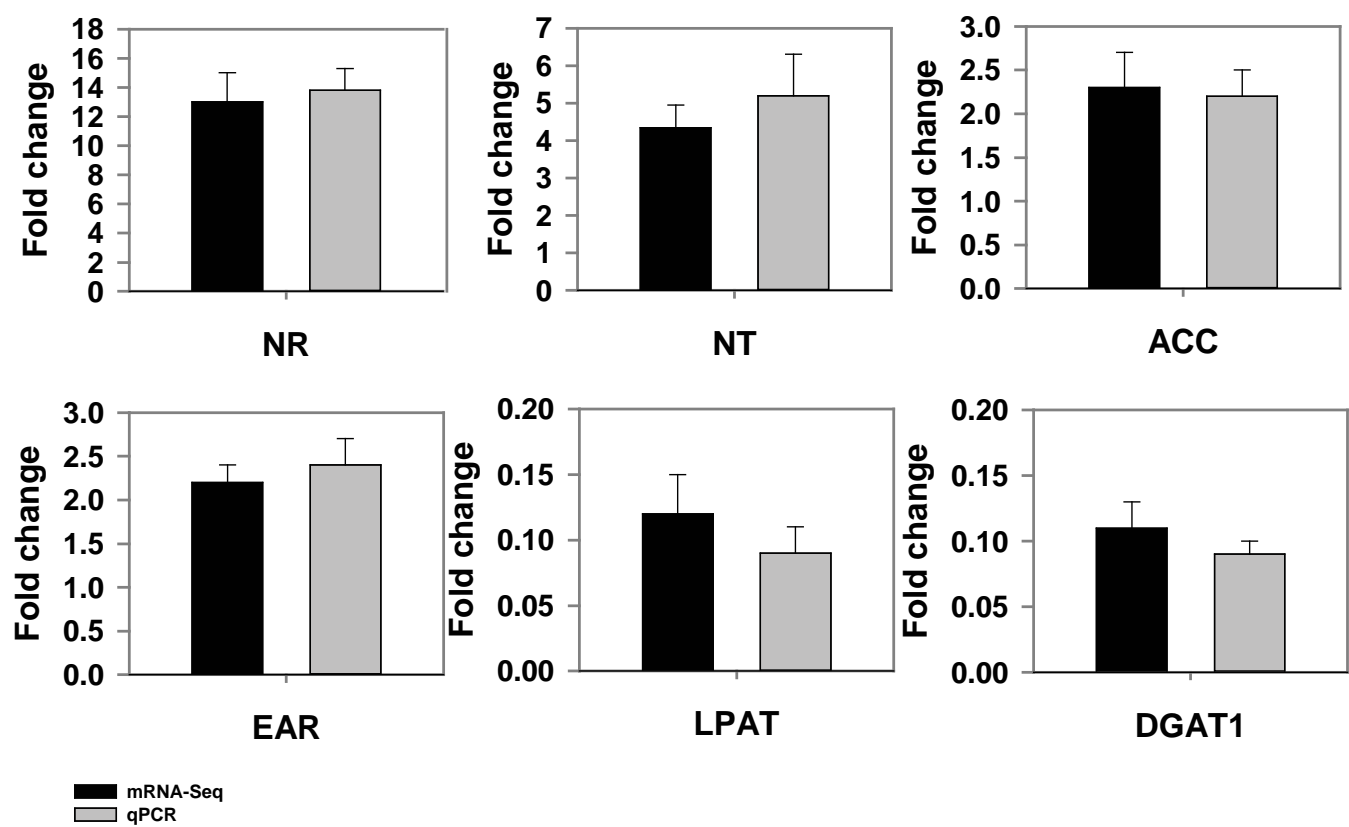

Supplement: S4 Fig — Species distribution of the total homologous sequences was calculated with match (with a cut-off E-value of 1.0E-5) in NR database. (PDF) [file pone.0259833.s004.pdf]

Figure S5

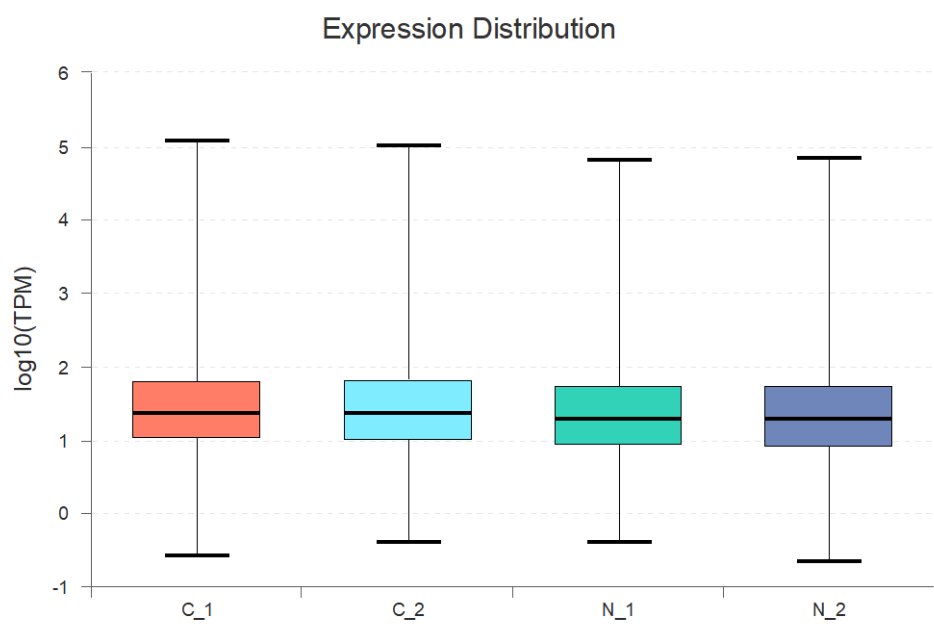

Supplement: S5 Fig — Species distribution of the total homologous sequences was calculated with match (with a cut-off E-value of 1.0E-5) in Nr database. (PDF) [file pone.0259833.s005.pdf]

Figure S6

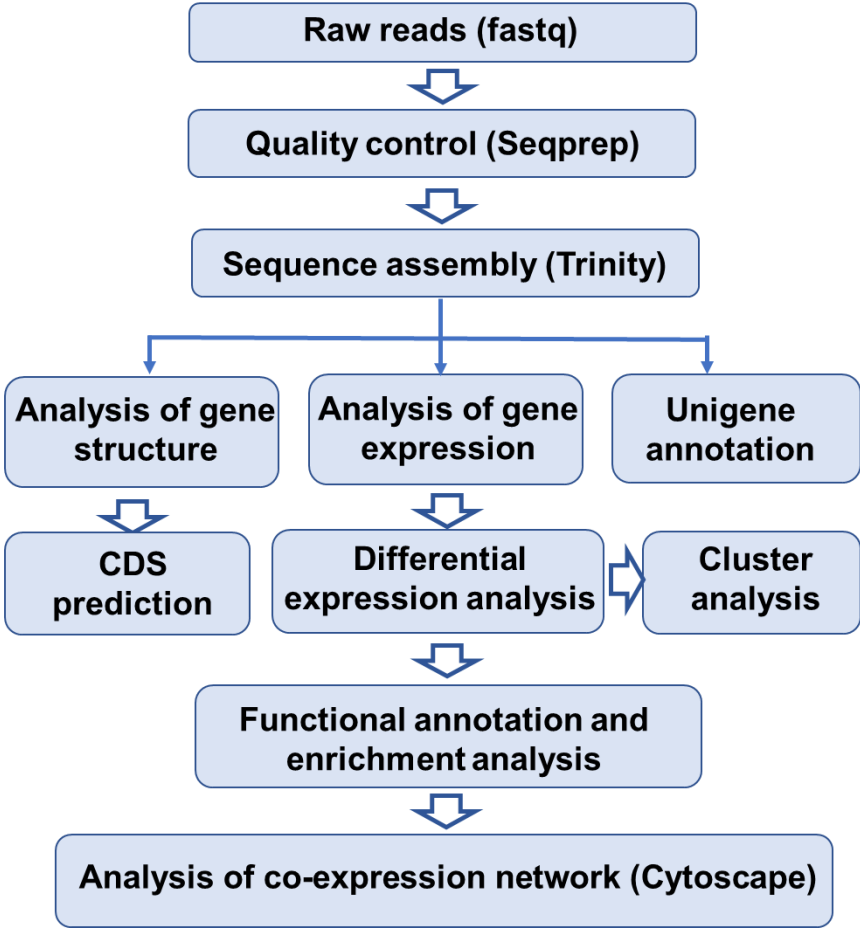

Supplement: S6 Fig — (PDF) [file pone.0259833.s006.pdf]
