# Supplementary material for: Transcriptomic survey reveals multiple adaptation mechanisms in response to nitrogen deprivation in marine Porphyridium cruentum
Source: PLoS One. 2021 Nov 18;16(11):e0259833. doi: 10.1371/journal.pone.0259833 (PMC8601545; doi:10.1371/journal.pone.0259833)
Supplement: S1 Table — (DOCX) [file pone.0259833.s007.docx]

**Table S1. De novo assembly length distribution of transcripts in *P.*** ***cruentum.***

| Length (bp) | Number of Unigene | Percent of Unigene |
| --- | --- | --- |
| 0~500 | 1876 | 23% |
| 501~1000 | 1264 | 15% |
| 1001~1500 | 1050 | 13% |
| 1501~2000 | 955 | 12% |
| 2001~2500 | 709 | 9% |
| 2501~3000 | 641 | 8% |
| 3001~3500 | 456 | 6% |
| 3501~4000 | 352 | 4% |
| 4001~4500 | 231 | 3% |
| >4500 | 710 | 9% |
