# Supplementary material for: Transcriptomic survey reveals multiple adaptation mechanisms in response to nitrogen deprivation in marine Porphyridium cruentum
Source: PLoS One. 2021 Nov 18;16(11):e0259833. doi: 10.1371/journal.pone.0259833 (PMC8601545; doi:10.1371/journal.pone.0259833)
Supplement: S2 Table — (DOCX) [file pone.0259833.s008.docx]

**Table S2. Primer sequences paired used in real time-qPCR**

| Gene ID | Gene name | Forward primer (5’-3’) | Reverse primer (5’-3’) |
| --- | --- | --- | --- |
| TRINITY_DN4606_c0_g2 | Actin | CTTGTAAATTTCGTCATGCGG | TTCAATTCAAAGAAGAAGATCCA |
| TRINITY_DN20_c0_g2 | Nitrate reductase (NR) | TGCGAGTCTAATGGACTCATCCAT | GAAAGCATGGGACCTGGCAGAG |
| TRINITY_DN867_c0_g1 | Nitrate transporter (NT) | TTCCAGAGGAAGAGCAATGGCT | CTTTGACGCAGAGATTGATTGG |
| TRINITY_DN1906_c0_g1 | Acetyl-CoA carboxylase (ACC) | GTGGTACCGCGTGATTGTGGC | ACGGATGCCCGTACTCGACCAG |
| TRINITY_DN1209_c0_g1 | Enoyl-ACP reductase (EAR) | CGGCTTCCAAGCAGAATGAAGAG | GGCTGCGTAAAGCGCCACATTCT |
| TRINITY_DN3436_c0_g1 | Lysophosphatidic acid acyltransferase (LPAT) | ATGATCTCCTACCTCGAAGGCACGC | GGTATTCTGCGCTGGTGAACATGTATC |
| TRINITY_DN756_c0_g2 | Diacylglycerol acyltransferase 1 (DGAT1) | TTATGCTGCCGGTGCTCATGCACT | TGCGATTCGACGAAAATATGCCGTA |
